# Clinical Features and Molecular Analysis of Hb H Disease in Taiwan

**DOI:** 10.1155/2014/271070

**Published:** 2014-08-28

**Authors:** Yu-Hua Chao, Kang-Hsi Wu, Han-Ping Wu, Su-Ching Liu, Ching-Tien Peng, Maw-Sheng Lee

**Affiliations:** ^1^Institute of Medicine, Chung Shan Medical University, No. 110, Section 1, Chien-Kuo N. Road, Taichung 402, Taiwan; ^2^Department of Pediatrics, Chung Shan Medical University Hospital, Taichung, Taiwan; ^3^School of Medicine, Chung Shan Medical University, Taichung, Taiwan; ^4^School of Chinese Medicine, China Medical University, Taichung, Taiwan; ^5^Department of Hemato-Oncology, Children's Hospital, China Medical University Hospital, China Medical University, Taichung, Taiwan; ^6^Department of Pediatrics, Buddhist Tzu Chi General Hospital, Taichung Branch, Taichung, Taiwan; ^7^Department of Biotechnology and Bioinformatics, Asia University, Taichung, Taiwan; ^8^Department of Obstetrics and Gynecology, Chung Shan Medical University Hospital, Taichung, Taiwan

## Abstract

Thalassemia is highly prevalent in Taiwan, but limited data are available about the association between genotypes and clinical manifestations in Taiwanese patients with Hb H disease. Here, we studied α-globin gene abnormalities and clinical features in Taiwanese patients with Hb H disease. Of the 90 patients, sixty-four (71.1%) were deletional and twenty-six (28.9%) were nondeletional Hb H disease. The (- -^SEA^) type of *α* ^0^-thalassemia mutation was detected in the majority of patients (>95%). The most common genotype was (- -^SEA^/-*α* ^3.7^), followed by (- -^SEA^/*α* ^cs^ *α*). After further investigation of the genotype-phenotype correlation in 68 patients, we found that patients with nondeletional Hb H disease had more severe clinical features than those with deletional Hb H disease, including younger age at diagnosis, more requirement of blood transfusions, and larger proportion of patients with splenomegaly, hepatomegaly or jaundice. This is probably a consequence of the lower hemoglobin levels and the higher Hb H levels. The clinical severity was highly variable even among patients with an identical genotype, and the diversity was much more profound among patients with (- -/*α* ^cs^ *α*) genotype. Therefore, predicting the phenotype directly from the genotype in Hb H disease remains relatively difficult in Taiwan.

## 1. Introduction

The α-thalassemia, arising from deletions or mutations of α-globin genes, is the most common inherited disease of hemoglobin synthesis in the world, especially in the Far East [1]. Three of the four α-globin genes are affected in patients with Hb H disease, which can show a wide spectrum of clinical phenotypes, ranging from no symptoms, to mild anemia with only occasional transfusions, to severe anemia and hemolysis with hepatosplenomegaly needing frequent transfusions, and even to fatal hydrops fetalis syndrome [2–4]. Due to the high carrier rate of approximately 5–7% of α-thalassemia [5], Hb H disease is not rare in Taiwan.

Due to the wide clinical spectrum of Hb H disease and the increased life expectancy in these patients, an individualized treatment approach is needed. Information about the genotype-phenotype correlation is thought important to assess patients with Hb H disease and beneficial for optimal patient management [6–8]. However, comprehensive clinical and molecular analysis in patients with Hb H disease in Taiwan is limited. Therefore, we assessed α-globin genotypes in 90 patients with diagnosis of Hb H disease in this study. The correlation between genotype and phenotype was further analyzed in 68 patients who received regular follow-ups at our hospitals.

## 2. Materials and Methods

From 1999 to 2013, ninety patients with diagnosis of Hb H disease by DNA analysis in the China Medical University Hospital were enrolled. These patients were referred to our laboratory for DNA analysis due to abnormal results of hematological studies and hemoglobin electrophoresis. Age of these patients ranged from 1.7 to 63.4 years. There were 47 males and 43 females. The majority of patients came from the middle part of Taiwan. The study was approved by the Institutional Review Board of the China Medical University Hospital.

All of the patients were initially presented with microcytic anemia, and hematological data were determined with an automated blood cell counter (Sysmex XE-2100 with SP-1000i series; Sysmex, Kobe, Japan). Hemoglobin analysis was performed with electrophoresis by automated high-performance liquid chromatography (PRIMUS CLC385; Primus Corporation, Kansas, MO, USA) according to manufacturer's instructions.

Genomic DNA was extracted from peripheral blood leukocytes using a commercially available kit (GE Healthcare, Buckinghamshire, UK). Identification of α-globin gene was done using allele-specific polymerase chain reaction (PCR) and direct sequencing analysis of α-globin gene. We designed primer sets on HBA1 (NM_000558.3) and HBA2 (NM_000517.3) DNA sequences. Mutations were detected by 11 pairs of primers using gap-PCR and PCR-restriction fragment length polymorphism-based methods, which were developed by Liu el al. [9, 10]. Certain α-globin gene mutations which are highly prevalent in Taiwan can be determined, including α^0^-thalassemia mutations (–^SEA^, –^Fil^, and –^THAI^), α^+^-thalassemia mutations (-α^3.7^ and -α^4.2^), the Constant Spring variant (*α* ^CS^α; TAA → CAA at codon 142 of *α*_2_ gene), and the Quong Sze variant (*α* ^QS^α; CTG → CCG at codon 125 of *α*_2_ gene). Unknown mutations were characterized by direct sequencing of the PCR-amplified product of *α*_2_- and *α*_1_-globin genes.

Clinical features were reviewed from medical records in 68 patients who received regular followups at the China Medical University Hospital or the Chung Shan Medical University Hospital, which are the main hospitals for the care of thalassemic patients in Taiwan. Complete physical examination and laboratory analysis of complete blood count and serum biochemical values were done every 3 months. In patients with abnormal results, such as splenomegaly, hepatomegaly, or jaundice, abdominal ultrasonography was performed every year thereafter. The size of spleen and liver was further measured by ultrasonography, and an age and gender matched reference range was used for the diagnosis of splenomegaly and hepatomegaly [11]. Clinical data were collected up to the last followup on May 31, 2014. Statistical analysis was performed with the SPSS 16.0 software program. Student's *t*-test was used to compare the continuous data, and Pearson chi-square or Fisher exact test was for categorical variables. The statistical value of *P* < 0.05 was considered to be significant.

## 3. Results

### 3.1. Genotypes of Hb H Disease

Table 1 shows the frequencies of various α-globin genotypes among the 90 Taiwanese patients with Hb H disease. Deletional Hb H disease is defined as a deletion removing both α-globin genes on one chromosome 16 plus a deletion removing only a single α-globin gene on the other chromosome 16, and nondeletional Hb H disease is a deletion removing both α-globin genes on one chromosome 16 plus an α^+^-thalassemia point mutation or a small insertion/deletion involving either the *α*_2_- or *α*_1_-globin gene on the other chromosome 16 [2, 12, 13]. Sixty-four (71.1%) were deletional Hb H disease and twenty-six (28.9%) were nondeletional Hb H disease. The (–^SEA^) type of α^0^-thalassemia mutation was detected in the majority of patients (>95%), and the (–^Fil^) type of α^0^-thalassemia mutation was detected in the remaining patients. There was no patient with the (–^THAI^) deletion in the present study. Among patients with deletional Hb H disease, the rightward deletion of 3.7 kb (-α^3.7^) was the most common type of mutation (71.2%), followed by the leftward deletion of 4.2 kb (-α^4.2^) and the G-Taichung variant with the 4.2 kb deletion (-*α* ^4.2  G-Taichung^). Among patients with nondeletional Hb H disease, most patients had the Constant Spring variant (84.6%), and the remaining patients had the Quong Sze variant. There was no patient with a compound heterozygosity for a nondeletional α^0^-thalassemia mutation and a nondeletional α^+^-thalassemia mutation in the present study.

### 3.2. Clinical Features and Their Association with Genotypes

A total of 90 patients with Hb H disease were enrolled for genotype study. Among them, sixty-eight patients who received regular follow-ups at our hospitals were further investigated the correlation between genotype and phenotype. The other 22 patients were excluded because of no sufficient clinical information available. Compared with patients with deletional Hb H disease, patients with nondeletional Hb H disease had more severe clinical features, including a younger age at diagnosis, more requirement of blood transfusions, and a larger proportion of patients with splenomegaly, hepatomegaly, or jaundice. Additionally, we found that the occurrence of splenomegaly could be an important indicator of clinical significance (see Table S1 in Supplementary Material available online at http://dx.doi.org/10.1155/2014/271070). In the present study, patients with hepatomegaly or jaundice always had splenomegaly concomitantly. Among the 14 patients with nondeletional Hb H disease who had splenomegaly, nearly all also had jaundice and half of them had hepatomegaly. Two patients with deletional Hb H disease suffered from cholecystitis with gallstones, and both received cholecystectomy during the episodes. Asymptomatic gallstones were accidentally found by abdominal ultrasonography in 5 patients with nondeletional Hb H disease, but none received cholecystectomy. Two patients experienced thrombotic events of lower legs in the fourth decade of life, and their genotypes were (–^SEA^/-*α* ^4.2  G-Taichung^) and (–^SEA^/*α* ^CS^α). None of the 68 patients had leg ulcers. Growth and pubescence were normal in all patients.

At diagnosis, patients with nondeletional Hb H disease had lower hemoglobin levels, higher MCV levels, and higher proportions of Hb H. They also had higher serum ferritin levels. In all patients with deletional Hb H disease, serum ferritin levels were not more than 800 ng/mL. Because glucose-6 phosphate dehydrogenase deficiency is also highly prevalent in Taiwan and can aggravate clinical manifestations in patients with Hb H disease, all patients were screened for the deficiency. Only 3 patients were positive for the deficiency, but none of them exhibited more severe anemia.  Table 2 summarizes the clinical and hematological features of these patients according to their genotypes.

## 4. Discussion

The incidence of genetic subtypes of Hb H disease varies greatly in different ethnic groups. The proportion of nondeletional Hb H disease was as high as more than 50% in Thailand, but as low as less than 20% in Cyprus and Sardinia [12, 14–16]. Of the 90 Taiwanese patients with Hb H disease in the present study, the nondeletional genotype accounted for 28.9%. The incidence of the Constant Spring variant was higher among patients with nondeletional Hb H disease in this study (84.6%) compared with Chen et al.'s report from Hong Kong [13]. The majority of patients with Hb H disease had the (–^SEA^) type of α^0^-thalassemia mutation, and the most common genotype was (–^SEA^/-α^3.7^), followed by (–^SEA^/*α* ^CS^α). In addition to the 90 patients with Hb H disease, we detected 12 patients with *β*-thalassemia associated with Hb E, which is the second common cause of thalassemia intermedia in Taiwan.

The diversity of clinical and hematological features was noted. We confirmed that patients with nondeletional Hb H disease had more severe clinical manifestations. Several possible mechanisms have been proposed to explain why synthesis of normal α-globin chains decreases in patients with nondeletional Hb H disease, including loss of compensation from the “healthy” α-globin gene and interference of transcription by the mutant α-globin gene [12, 17]. More than half of these patients with nondeletional Hb H disease had a history of blood transfusions, and some of them even were transfusion dependent, whereas many patients with deletional Hb H disease were first diagnosed after infection-induced hemolysis or during health assessment or pregnancy, therefore older in age at diagnosis among these patients. Less than one-quarter of patients with deletional Hb H disease had history of transfusions, and the majority received less than 3 transfusions. None were transfusion dependent. Splenomegaly, hepatomegaly, and jaundice were also more common in patients with nondeletional Hb H disease. It is probably due to the severity of anemia and propensity towards hemolysis in these patients as a consequence of the lower hemoglobin levels and higher Hb H levels. Additionally, it was interesting to find that two patients with deletional Hb H disease received cholecystectomy due to acute cholecystitis, whereas about 20% of patients with nondeletional Hb H disease had silent gallstones, but this did not develop into acute or chronic cholecystitis. However, we did not detect any factors contributing to the high prevalence of gallstones, but low incidence of symptomatic cholecystitis in these patients with nondeletional Hb H disease.

In contrast to the report of Chen et al. which found that the G-Taichung variant would aggravate the clinical symptoms in patients with Hb H disease [18], the 8 patients with (–/-*α* ^4.2  G-Taichung^) genotype in this study were clinically similar to those with deletional Hb H disease, such as (–/-α^3.7^) or (–/-α^4.2^) genotypes. These patients had less severe clinical features compared with those with nondeletional Hb H disease. Patients with the Quong Sze variant are thought to be more prone to hemolysis because the intracellular aggregates of hyperunstable variant of α-globin chains might cause additional membrane damage and dysfunction [2]. But the severity of clinical features in the 4 patients with (–^SEA^/*α* ^QS^α) genotype was similar to that with (–^SEA^/*α* ^CS^α) genotype in this study. Of importance, we found that the clinical severity was highly variable even among patients with an identical genotype. It may relate to the complex interaction between environmental and genetic factors. The diversity was found much more profound among those with (–/*α* ^CS^α) genotype. Therefore, predicting the phenotype directly from the genotype in Hb H disease remains relatively difficult in Taiwan.

## Supplementary Material

The occurrence of splenomegaly could be an important indicator of clinical significance. In the present study, patients with hepatomegaly or jaundice always had splenomegaly concomitantly. Among the 14 patients with nondeletional Hb H disease who had splenomegaly, nearly all of them also had jaundice and half of them had hepatomegaly.

## Figures and Tables

**Table 1:** α-Globin genotypes of the 90 patients with Hb H disease in Taiwan.

α-Globin genotype	Number of patients	%
Deletional Hb H disease		
- -^SEA^/-α^3.7^	43	47.8
- -^SEA^/-α^4.2^	10	11.1
- -^SEA^/-α^4.2 G-Taichung^	7	7.8
- -^Fil^/-α^3.7^	3	3.3
- -^Fil^/-α^4.2 G-Taichung^	1	1.1
Nondeletional Hb H disease		
- -^SEA^/α^CS^α	22	24.5
- -^SEA^/α^QS^α	4	4.4
Total	**90**	**100**

**Table 2:** Comparison of clinical features between patients with deletional and nondeletional Hb H disease in Taiwan.

	Deletional Hb H disease	Nondeletional Hb H disease	*P* value
	(*n* = 44)	(*n* = 24)	
Initial manifestations			
Age at diagnosis (year)	1.7–63.4 (median 24.0)	3.0–36.9 (median 10.4)	0.040∗
Gender	21 males, 23 females	15 males, 9 females	0.261
Laboratory data at diagnosis			
Hb (g/dL)	5.5–11.5 (median 9.1)	4.6–12.1 (median 8)	0.001∗
MCV (fL)	43.9–75.1 (median 60)	63.0–78.1 (median 68.8)	<0.001∗
MCH (pg)	15.1–28.0 (median 17.5)	16.8–22.4 (median 18.3)	0.568
MCHC (g/dL)	15.3–33.7 (median 29.0)	19.1–29.4 (median 25.9)	0.740
Hb H (%)	0.7–29.7 (median 5.9)	6.3–35.5 (median 12.3)	<0.001∗
Follow-up clinical features			
History of blood transfusions	10 (22.7%)	14 (58.3%)	0.003∗
Splenomegaly	9 (20.5%)	14 (58.3%)	0.002∗
Hepatomegaly	3 (6.8%)	7 (29.2%)	0.013∗
Jaundice	4 (9.1%)	13 (54.2%)	<0.001∗
Gallstones	2 (4.5%)	5 (20.8%)	0.035∗
Cholecystitis	2 (4.5%)	0	0.289
Splenectomy	4 (9.1%)	5 (20.8%)	0.172
Cholecystectomy	2 (4.5%)	0	0.289
Thrombotic event	1 (2.3%)	1 (4.2%)	0.659
Leg ulcers	0	0	—
Growth retardation	0	0	—
Delay of pubescence	0	0	—
Serum ferritin level (ng/mL)	40.2–699.0 (median 142.5)	40.3–1619.0 (median 210.7)	0.001∗

*P < 0.05.

## References

[1] Weatherall DJ (2012). The definition and epidemiology of non-transfusion-dependent thalassemia. *Blood Reviews*.

[2] Chui DHK, Fucharoen S, Chan V (2003). Hemoglobin H disease: Not necessarily a benign disorder. *Blood*.

[3] Taher A, Isma'eel H, Cappellini MD (2006). Thalassemia intermedia: revisited. *Blood Cells, Molecules, and Diseases*.

[4] Musallam KM, Rivella S, Vichinsky E, Rachmilewitz EA (2013). Non-transfusion-dependent thalassemias. *Haematologica*.

[5] Chang JG, Liu HJ (1995). Molecular diagnosis of thalassemia in Taiwan.. *Kaohsiung Journal of Medical Sciences*.

[6] Vichinsky E (2012). Advances in the treatment of alpha-thalassemia. *Blood Reviews*.

[7] Fucharoen S, Viprakasit V (2009). Hb H disease: clinical course and disease modifiers. *Hematology*.

[8] Peng CT, Chang JS, Wang LY (2009). Update on thalassemia treatment in taiwan, including bone marrow transplantation, chelation therapy, and cardiomyopathy treatment effects. *Hemoglobin*.

[9] Liu YT, Old JM, Miles K, Fisher CA, Weatherall DJ, Clegg JB (2000). Rapid detection of α-thalassaemia deletions and α-globin gene triplication by multiplex polymerase chain reactions. *British Journal of Haematology*.

[10] Chang JG, Lee LS, Lin CP, et al (1991). Rapid diagnosis of α-thalassemia-1 of southeast Asia type and hydrops fetalis by polymerase chain reaction. *Blood*.

[11] Dhingra B, Sharma S, Mishra D, Kumari R, Pandey RM, Aggarwal S (2010). Normal values of liver and spleen size by ultrasonography in Indian children. *Indian Pediatrics*.

[12] Laosombat V, Viprakasit V, Chotsampancharoen T (2009). Clinical features and molecular analysis in Thai patients with HbH disease. *Annals of Hematology*.

[13] Chen FE, Ooi C, Ha SY (2000). Genetic and clinical features of hemoglobin H disease in Chinese patients. *New England Journal of Medicine*.

[14] Charoenkwan P, Taweephon R, Sae-Tung R, Thanarattanakorn P, Sanguansermsri T (2005). Molecular and clinical features of Hb H disease in northern Thailand. *Hemoglobin*.

[15] Baysal E, Kleanthous M, Bozkurt G (1995). α-Thalassaemia in the population of Cyprus. *British Journal of Haematology*.

[16] Origa R, Sollaino MC, Giagu N (2007). Clinical and molecular analysis of haemoglobin H disease in Sardinia: haematological, obstetric and cardiac aspects in patients with different genotypes. *British Journal of Haematology*.

[17] Higgs DR, Weatherall DJ (2009). The Alpha thalassaemias. *Cellular and Molecular Life Sciences*.

[18] Chen T, Liu T, Chang C, Chang J, Tsai H, Lin S (2002). PCR-based analysis of α-thalassemia in Southern Taiwan. *International Journal of Hematology*.

