# Supplementary material for: Clinical Features and Molecular Analysis of Hb H Disease in Taiwan
Source: Biomed Res Int. 2014 Aug 28;2014:271070. doi: 10.1155/2014/271070 (PMC4163353; doi:10.1155/2014/271070)
Supplement: Supplementary file 1 — The occurrence of splenomegaly could be an important indicator of clinical significance. In the present study, patients with hepatomegaly or jaundice always had splenomegaly concomitantly. Among the 14 patients with nondeletional Hb H disease who had splenomegaly, nearly all of them also had jaundice and half of them had hepatomegaly. [file 271070.f1.docx]

TABLE S1: Clinical information about splenomegaly and hepatomegaly of the 23 patients with Hb H disease who had splenomegaly^†^ in the present study.

| Patient no. | Gender | Age (years) | Spleen length^*^ (cm) | Splenectomy | Hepatomegaly^†^ | Liver length^*^ (cm) |
| --- | --- | --- | --- | --- | --- | --- |
| Deletional Hb H disease | | | | | | |
| D1 | F | 27.3 | 12.0 | Yes | No | 11.5 |
| D2 | F | 43.0 | 12.5 | Yes | Yes | 16.0 |
| D3 | M | 21.6 | 16.5 | No | Yes | 16.5 |
| D4 | F | 30.0 | 12.5 | Yes | No | 14.5 |
| D5 | F | 57.0 | 12.0 | No | Yes | 15.5 |
| D6 | M | 36.9 | 17.0 | No | No | 13.5 |
| D7 | M | 39.8 | 12.5 | Yes | No | 13.0 |
| D8 | F | 25.0 | 12.0 | No | No | 13.0 |
| D9 | F | 50.3 | 12.5 | No | No | 13.5 |
| Non-deletional Hb H disease | | | | | | |
| N1 | M | 18.7 | 15.0 | Yes | Yes | 16.0 |
| N2 | M | 17.8 | 12.0 | No | No | 11.0 |
| N3 | M | 16.3 | 16.5 | No | No | 10.5 |
| N4 | M | 19.1 | 12.5 | No | Yes | 15.0 |
| N5 | F | 22.1 | 12.5 | No | Yes | 15.5 |
| N6 | M | 19.0 | 19.0 | No | Yes | 16.0 |
| N7 | M | 13.0 | 14.0 | Yes | No | 8.9 |
| N8 | M | 29.2 | 12.0 | No | No | 10.0 |
| N9 | M | 39.2 | 12.0 | Yes | Yes | 15.5 |
| N10 | F | 39.0 | 13.0 | Yes | Yes | 16.0 |
| N11 | M | 13.0 | 14.0 | No | Yes | 16.0 |
| N12 | F | 39.1 | 13.0 | No | No | 12.0 |
| N13 | F | 20.9 | 14.0 | Yes | No | 12.5 |
| N14 | F | 30.5 | 12.5 | No | No | 12.0 |

F: female; M: male.

^*^The length of spleen and liver was measured by ultrasonograpy.

^†^An age and gender matched reference range was used for the diagnosis of splenomegaly and hepatomegaly [11].
